# Supplementary material for: Theoretical investigation of active listening behavior based on the echolocation of CF-FM bats
Source: PLoS Comput Biol. 2022 Oct 7;18(10):e1009784. doi: 10.1371/journal.pcbi.1009784 (PMC9581360; doi:10.1371/journal.pcbi.1009784)
Supplement: S1 Text — (PDF) [file pcbi.1009784.s002.pdf]

S1 Text. *Echo amplitude representation procedure with four omni-directional microphones*

As shown in Fig 2A, the target is located in the direction pointed to by the unit vector $\boldsymbol{n} = (n_x, n_y, n_z)$, and the distance from the origin r is sufficiently larger than the distance d between the directional ear and the speaker and the spacing δ_y, δ_z between the directional microphones. A sinusoidal sound wave of frequency f is emitted from the speaker and the reflected wave returns from the target. Assume that both of the directional ears are set in the original orientation expressed by the identity matrix, as shown in Fig 2B, and the reflection wave received by the speaker is given by $s(t) = A \sin 2\pi f t$. Using the position vectors $\boldsymbol{r}^l_{\pm\pm} = \left(0, \frac{d}{2} \pm \frac{\delta_y}{2}, \pm \frac{\delta_z}{2}\right)$, $\boldsymbol{r}^r_{\pm\pm} = \left(0, -\frac{d}{2} \pm \frac{\delta_y}{2}, \pm \frac{\delta_z}{2}\right)$ of the eight microphones and the assumption that $\delta_y, \delta_z, d \ll r$, the distances between the target and the microphones are calculated as

$$r + \Delta r^l_{\pm\pm},\ r + \Delta r^r_{\pm\pm}, \qquad (S1)$$

where

$$\Delta r^l_{\pm\pm} \simeq -n_y \frac{d}{2} \mp n_y \frac{\delta_y}{2} \mp n_z \frac{\delta_z}{2}, \qquad (S2)$$

$$\Delta r^r_{\pm\pm} \simeq n_y \frac{d}{2} \mp n_y \frac{\delta_y}{2} \mp n_z \frac{\delta_z}{2}. \qquad (S3)$$

The signal received by the left directional ear is given by

$$s^l(t) = s(t - \Delta t^l_{++}) + s(t - \Delta t^l_{\pm}) + s(t - \Delta t^l_{\mp}) + s(t - \Delta t^l_{--}) \qquad (S4)$$

with $\Delta t^l_{\pm\pm} = \Delta r^l_{\pm\pm}/c$, where c is the sound velocity. A simple calculation using trigonometric functions gives

$$S^l(t) = 4A \cos \frac{\pi n_y \delta_y}{\lambda} \cos \frac{\pi n_z \delta_z}{\lambda} \sin 2\pi f \left(t + \frac{n_y d}{2c}\right) \qquad (S5)$$

Similarly, the signal on the right side is given by

$$S^r(t) = 4A\cos\frac{\pi n_y \delta_y}{\lambda}\cos\frac{\pi n_z \delta_z}{\lambda}\sin 2\pi f\left(t - \frac{n_y d}{2c}\right) \tag{S6}$$

Note that the amplitudes of both $s^l(t)$, $s^r(t)$ are the same and the distance d only influences the phase of the received signal.

Let us consider one of the directional ears. When it has an orientation expressed by $L \in SO(3)$, we rotate the space by $L^{-1} = L^T$ and the situation becomes the same as above. Therefore, the amplitude of the received signal is $A_L = 4A\,cos\frac{\pi\tilde{n}_y\delta_y}{\lambda}\,cos\frac{\pi\tilde{n}_z\delta_z}{\lambda}$, where $L^T\boldsymbol{n} = \tilde{\boldsymbol{n}} = (\tilde{n}_x, \tilde{n}_y, \tilde{n}_z)$. A_L is always positive because δ_y and δ_z are slightly smaller than $\frac{\lambda}{2}$. The calculation process remains valid if the orientation varies with time, i.e., $L(t)$. The envelope of the received signal is then a positive function given by

$$S_{env}(t) = 4A\cos\frac{\pi\tilde{n}_y(t)\delta_y}{\lambda}\cos\frac{\pi\tilde{n}_z(t)\delta_z}{\lambda} \tag{S7}$$

where $L(t)^T\boldsymbol{n} = \tilde{\boldsymbol{n}}(t) = \left(\tilde{n}_x(t), \tilde{n}_y(t), \tilde{n}_z(t)\right)$.
