# Supplementary material for: Theoretical investigation of active listening behavior based on the echolocation of CF-FM bats
Source: PLoS Comput Biol. 2022 Oct 7;18(10):e1009784. doi: 10.1371/journal.pcbi.1009784 (PMC9581360; doi:10.1371/journal.pcbi.1009784)
Supplement: S2 Text — (PDF) [file pcbi.1009784.s003.pdf]

S2 Text. *Explanations for the evaluation function* $\boldsymbol{U_F}$ *and the degree of injection* $\boldsymbol{I}[\boldsymbol{F}]$.

In this supplement, we explain how the evaluation function and the degree of injection work using very simple examples. Let us consider four functions f_1, f_2, f_3, f_4 defined on the closed interval [-1, 1] as indicated in Fig 1. Note that the parameter ϵ of f_4 is a very small positive value.

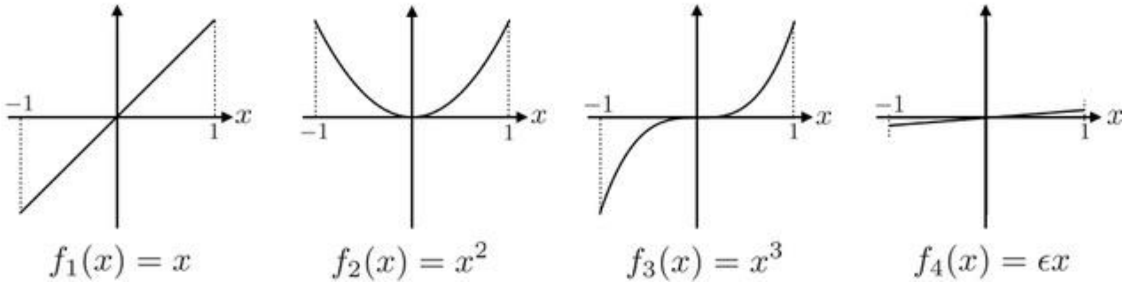

Fig 1. Various functions

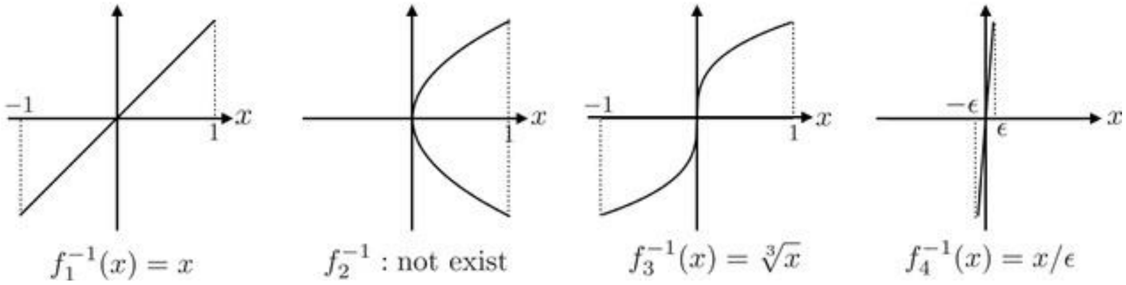

Fig 2. Inverse functions

Each graph in Fig 2 is just a mirrored image of the graph in Fig 1 with respect to $y = x$. As easily seen, only f_1 has well-behaved inverse function, while others do not. The function f_2 has no inverse function because it is not injective. The inverse function ${f_3}^{-1}$ eixsts, but is not well-behaved around 0. It is because f_3 degenerate at 0 (*i.e.* ${f_3}'(0) = 0$) , thus${f_3}^{-1}$ has a singularity at 0 (*i.e.*$({f_3}^{-1})'(0) = +\infty$) . Also, the inverse function

f_4^{-1} eixsts, but is not well-behaved. It is because f_4 nearly degenerates, thus f_4^{-1} is nearly singular. Both of f_3^{-1} and f_4^{-1} are very sensitive to the noise in the vicinity of 0.

Applying the definition (9) and (10) to these examples, the evaluation function and the degree of injection are written as

$$U_{F_i}(x) = \sup_{x\prime \neq x} \frac{|x - x'|}{|F_i(x) - F_i(x')|}$$

for *i=1,2,3,4*

$$I[F_i] = \int_{-1}^{1} U_{F_i}(x)\, dx.$$

These are calculated by hand as follows,

$$U_{F_1}(x) = \sup_{x\prime \neq x} \frac{|x - x\prime|}{|x - x\prime|} \equiv 1 \ \rightarrow \ I[f_1] = 1/2$$

$$U_{F_2}(x) = \sup_{x\prime \neq x} \frac{|x - x'|}{|x^2 - x'^2|} \equiv +\infty \ \rightarrow \ I[f_2] = 0$$

$$U_{F_3}(x) = \sup_{x\prime \neq x} \frac{|x - x'|}{|x^3 - x'^3|} \equiv \frac{4}{3x^2} \ \rightarrow \ I[f_3] = 0$$

$$U_{F_1}(x) = \sup_{x\prime \neq x} \frac{|x - x\prime|}{|\epsilon x - \epsilon x\prime|} \equiv \frac{1}{\epsilon} \ \rightarrow \ I[f_4] = \epsilon/2 \ \ll 1.$$

As easily seen in the calculation process, break of injection and degeneration (including nearly degenerated case) give infinite value or very large value to the evaluation function, consequently, makes the degree of injection zero or very small. If the degree of injection is not small, existence of well-behaved inverse function is guaranteed. Although these examples are demonstrated just for explanation, the essential reason why the degree of injection can evaluate the quality of inverse map is the same with much complicated higher dimensional case.
