# Supplementary material for: Theoretical investigation of active listening behavior based on the echolocation of CF-FM bats
Source: PLoS Comput Biol. 2022 Oct 7;18(10):e1009784. doi: 10.1371/journal.pcbi.1009784 (PMC9581360; doi:10.1371/journal.pcbi.1009784)
Supplement: S3 Text — (PDF) [file pcbi.1009784.s004.pdf]

S3 Text. *Expression of the spatial orientation of the directional ear.*

There are many ways to express the elements of $SO(3)$ using three parameters. We adopt the roll–pitch–yaw expression in this article. Let us consider a sequence of rotations from the original orientation shown in Fig 1B:

(1) Rotate around the yaw axis by an angle of θ_e;

(2) Rotate around the pitch axis by an angle of φ_e (elevation is taken in the positive direction, so φ_e is the inverse of the usual pitch angle);

(3) Rotate around the roll axis by an angle of ψ_e.

We then have the rotation matrix $L = R_z(\theta_e)\, R_y(-\varphi_e)\, R_x(-\psi_e)$, where $R_\alpha(\zeta)$ denotes a matrix that express a rotation around the α-axis by an angle of ζ.
