# Supplementary figures and images for: Theoretical investigation of active listening behavior based on the echolocation of CF-FM bats
Source: PLoS Comput Biol. 2022 Oct 7;18(10):e1009784. doi: 10.1371/journal.pcbi.1009784 (PMC9581360; doi:10.1371/journal.pcbi.1009784)

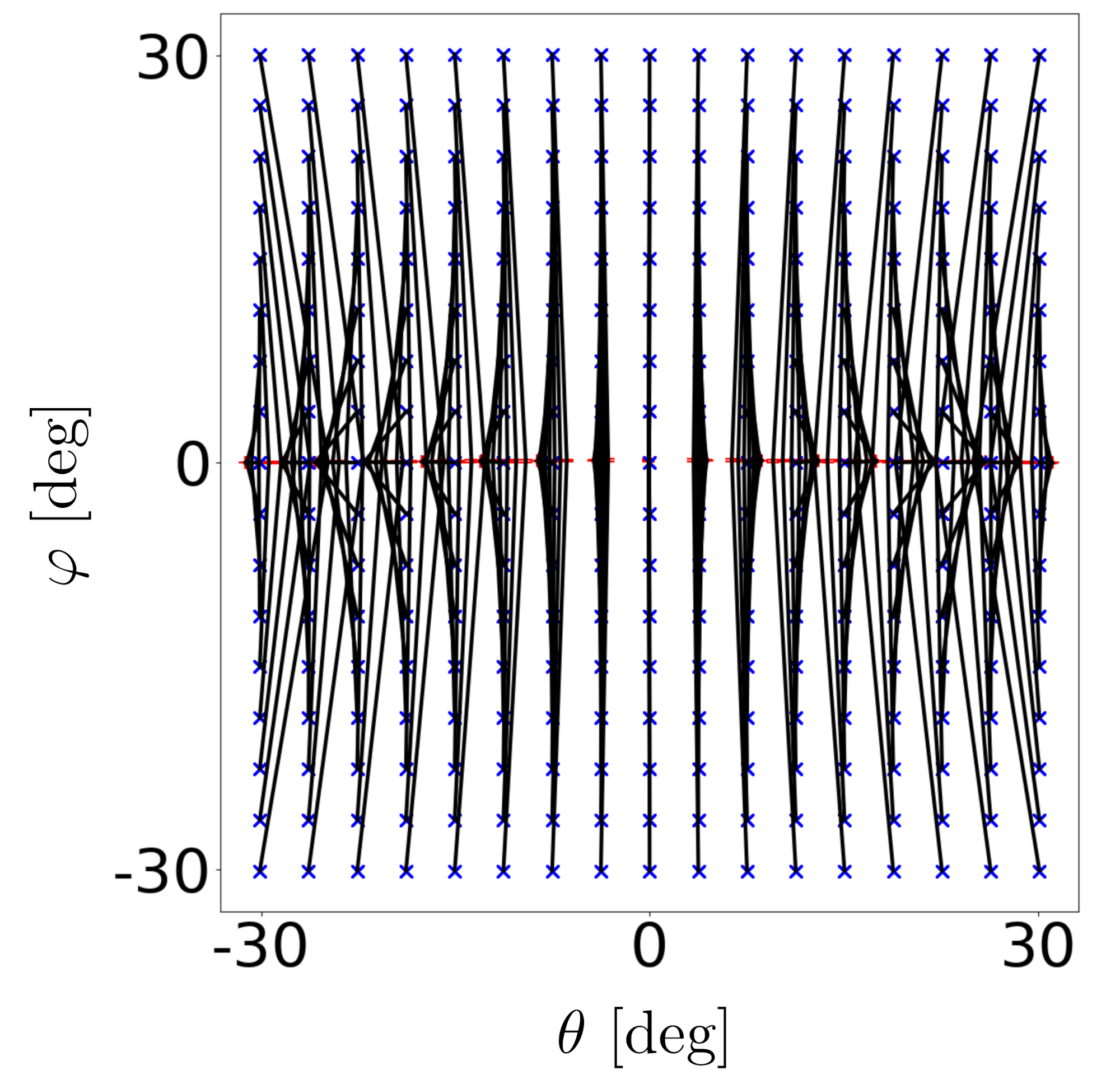

Supplement: S1 Fig — The ear motion condition was chosen as [ψel,r: 0, φel,r: 0, θel,r: CONST¯]. Blue ‘x’ markers indicate test data (θ, φ) and red ‘+’ markers indicate output data (θguess, φguess). Black lines denote the error lines connecting points (θ, φ) and (θguess, φguess). Each detection error line tends to stretch vertically, indicating that the elevation angle is difficult to detect while the azimuth angle can be accurately detected. (TIF) [file pcbi.1009784.s005.tif]
