# Supplementary material for: Theoretical investigation of active listening behavior based on the echolocation of CF-FM bats
Source: PLoS Comput Biol. 2022 Oct 7;18(10):e1009784. doi: 10.1371/journal.pcbi.1009784 (PMC9581360; doi:10.1371/journal.pcbi.1009784)
Supplement: Attachment — Submitted filename: response_letter_for_revision.docx [file pcbi.1009784.s006.docx]

*PLOS Computational Biology*

May 4, 2022

Dear Editor,

Re: Resubmission of manuscript reference No. PCOMPBIOL-D-21-02303

Please find attached a revised version of our manuscript “Theoretical investigation of active listening behavior based on the echolocation of CF-FM bats,” which we would like to submit for consideration for publication as an Original Article in *PLOS Computational Biology.*

The comments from the reviewers were highly insightful and enabled us to greatly improve the quality of our manuscript. The following pages present our point-by-point responses to each of the comments of the reviewers.

We hope that the revisions in the manuscript and our accompanying responses will be sufficient to make our manuscript suitable for publication in *PLOS Computational Biology.*

We look forward to hearing from you at your earliest convenience.

Yours sincerely,

Yasufumi Yamada (Corresponding author)

Program of Mathematical and Life Sciences, Hiroshima University.

C-623, Department of Sciences,1-3-2 Kagamiyama, Higashi-Hiroshima, Japan

E-mail: yasufumi.yamada1@gmail.com

**Editor Remarks:**

The reviewers are agreed that the paper is of interest, and appropriate for PLOS Computational Biology, but have substantial required changes. I therefore agree with their recommendation to request Major Revisions. Please pay attention to the reviewers' feedback. This includes fixing the data availability issue.

*Acknowledgment*:	We would like to thank the editor and reviewers for taking the time to examine our manuscript. We have made a significant effort to revise the manuscript according to the various comments made by the reviewers. We hope that our revised paper is suitable for publication in your esteemed journal. Our responses to the individual comments of the reviewers can be found below.

**Reviewer #1**

**Reviewer #1 Remarks:**

Hiraga et al. present an interesting theoretical study analyzing the benefits of ear movements on sound localization. The role of ear movements in sound localization is still not fully understood and this study takes us a step forward by testing different movement combinations. One of the interesting findings in the paper is that the pitch movement of the two ears should be in anti-phase which is what is observed in many bats. The study thus makes an important contribution to the question but unfortunately, the manuscript was very difficult to understand. Most of my comments below arise from a failure to understand the authors. I will give several examples:

*Acknowledgment*:	Thank you for recognizing the value of our theoretical findings regarding the ear movements necessary for direction detection using ultrasound. We expect our theoretical findings will be of great interest to behavioral scientists. We have made a significant effort to revise the manuscript according to your suggestions. In particular, the Methods section has been carefully revised to provide a clearer explanation.

**General Comments:**

Comment 1:	The authors provide a long section about general estimation in the methods titled: evaluation function and degree of injection, but it was very difficult for me to read how this method connects to the machine learning and why are there two different methods of evaluation.

*Revised point:*	We regret that our mathematically strict description of the problem has caused some confusion. Our aim was to construct a general mathematical framework that describes and evaluates the sensing process, particularly the active sensing process. In case of active sensing, observation data are usually a function of time, which requires some more advanced mathematics. This is why we prepared a special mathematical section.

To highlight the significance of this mathematical description, we have modified the start of this section as follows:

pp.11, Lines 255–260:	*In this section, we prepare a general mathematical framework for describing and evaluating the sensing process, particularly the active sensing process. The sensing process consists of two phases. In the first phase, we obtain data from the objective system, and in the second phase, we identify the variable of interest based on the obtained data. To evaluate the design of the sensing process, we introduce a general evaluation function and the degree of injection index.*

To answer the question about the two different methods requires a mathematical explanation. Therefore, the answer is stated in the response to **comment 2**.

Comment 2:	Moreover, I did not understand that use of ‘X’ or ‘n’ in the injection function. Shouldn’t it rely on Y only? Aren’t we aiming to predict similarity in x based on similarity in y? I feel that there is much to clarify.

*Revised point:*	To explain how the mathematical framework works, consider the following simple example.

Example: Detection of velocity of moving object using Doppler shift of reflected ultrasonic wave.

In this simple example, *X* is a set of velocities and *Y* is a set of frequencies. *F* is explicitly written as $f=F\left( v \right)=f_{0}(c+v)/(c-v)$ according to the formula of Doppler shift (physical process). In this case, $F^{-1}$ is obtained as $v=F^{-1}\left( f \right)=c(f-f_{0})/(f+f_{0})$ by an elementary calculation (computational process). Note that the set *X* is absolutely necessary for describing the observation process mathematically. The setting $F:X\to Y$ is essential.

In our problem, *X* is the set of directions *n* (or equivalently, pairs of azimuth and elevation angle) and *Y* is the set of temporal data of ILD (function of time). *F* can be explicitly written using Eqs. (1) and (2), but $F^{-1}$ cannot, because the observed data are not simple values, but are a function of time. The computational process for detecting the angle pairs from the observed temporal data is significantly more difficult than the above simple example. To compute $F^{-1}$, we adopted supervised machine learning.

Your question is “Aren’t we aiming to predict similarity in *x* based on similarity in *y*? I feel that there is much to clarify.”

If the similarity between the observed data means the similarity between the variables to be detected, the observation process is well designed. However, this is not always true. To “judge” whether the observation process is well designed or not, we developed the concepts of an evaluation function and the degree of injection.

To explain the usefulness of the evaluation function and degree of injection, we have added a supplemental section (**S2 text**) and the following sentence.

pp.13, Lines 294-295: *To clarify the meaning of* $U_{F}\left( x \right)$ *and* $I\left[ F \right]$*, we present a full description in the supplemental text (see* ***S2 Text****).*

Machine learning is a method of “constructing” the inverse map $F^{-1}$, and once this has been constructed, we can evaluate the accuracy of the sensing process. However, the construction of $F^{-1}$ by machine learning is computationally intensive, while the degree of injection is much simpler to calculate. Thus, it is essential that the index can evaluate the sensing process without constructing $F^{-1}$.
The reason why both methods are demonstrated in our article is that we wish to demonstrate how well our new index works.

Based on these responses, we changed the title of the section “Evaluation of localization performance by supervised machine learning” to “Construction of the inverse map using supervised machine learning” (pp.14, Lines 324-325) and have added NOTE 2 at the end of the section:

pp. 16, Lines 363-371: *NOTE 2: Once the inverse map (or pseudo-inverse map) has been constructed, we can evaluate whether it is well-behaved using* $E\left[ M \right]$ *in Eq. (11). Thus, the degree of injection* $I\left[ M \right]$ *appears to be unnecessary. However, the construction of* $M^{-1}$ *through machine learning is computationally intensive, whereas the degree of injection is relatively simple to calculate. Therefore, considering the computational cost (which is essential in more complicated problems), it is sensible to construct the inverse map* $M^{-1}$ *only for promising motions* $M$ *identified by the degree of injection* $I\left[ M \right]$*. At the same time, we construct* $M^{-1}$ *and compare* $E\left[ M \right]$ *and* $I\left[ M \right]$*for some typical motions as a means of demonstrating that our new index* $I\left[ M \right]$*works well.*

Comment 3:	I also did not understand why are the microphones placed in 4 discrete locations if this is a simulation? Why are they not continuous?

*Response:*	Thank you for this important comment. Hearing directivity patterns have been measured in several species of bats, as described in the Methods section. However, the directivity pattern is different in every species of bat, so we could not determine the common characteristics beyond the anisotropic directivity pattern. Therefore, to construct the anisotropic directivity pattern through a simple method, the microphones were placed in four discrete locations. This is the popular beamforming method from the engineering field. This quadruple microphone method makes it easy to customize the hearing directivity pattern in the horizontal and vertical directions by adjusting the horizontal and vertical spacing between the microphones (i.e., by adjusting $\delta_{y}$ and $\delta_{z}$ as shown in Fig 2B).

Comment 4:	L128 ‘Based on these findings, asymmetrical ear motions were embedded in bat mimicking Simulations’ – perhaps it is a problem of the English but it is not clear what do the authors mean by asymmetric? Do they mean anti-phase?

*Revised point:*	We apologize for the ambiguous and incorrect English. We have modified this sentence as follows:

pp. 7, Lines 165–167: *Based on these findings, anti-phase pitch motions were assumed in the bat-mimicking simulations.*

**Scientific comments:**

Comment 5:	I had two scientific comments:
1) The model completely ignores the spectral filtering that is happening in the ear. What about the HRTF? And what about moving / fluttering prey which is what these bats typically hunt?

*Revised point:*	Thank you for your important comment. As you point out, the spectral filtering function of the ear is an important feature when broadband FM signals are used for direction detection. However, in this article, we assumed that the reflective sound source direction could be detected by the amplitude modulation of the CF component. Following your comment, we will investigate the utility of a spectral filtering function in future research.
We agree that much more complex hearing directivity patterns, as employed by actual bats, should be discussed alongside our findings. Spectral cues are also important factors to investigate the direction detection mechanism employed by bats. Therefore, we have added a new paragraph in the Discussion section:

pp. 24, Lines 556-572: *In our simulations, a simple anisotropic directivity pattern was assumed for hearing. However, actual measurements of bats have shown that the hearing directivity pattern is more complex in periphery regions [17, 18, 30, 34]. Another measurement study showed that the hearing directivity pattern of bats is deformed by the bending of the soft material of the ears [30]. Such a complex hearing directivity pattern has not been investigated in the present study. There is the possibility to deregulate the ear motion conditions by implementing more precise hearing directivity pattern in bats. If so, this would provide a better understanding of the directivity formation effect in bats’ pinnae. From another perspective, it has been suggested that the ear movements produce detectable Doppler shifts, with the time-frequency Doppler shift signatures able to encode the sound source direction in an orderly fashion [20]. If we clarify the necessary conditions of ear movements for direction detection with Doppler shift signatures using a similar analysis framework, we could compare the differences in motion conditions between ILD-based and Doppler shift-based direction detection. Such a comparison could be expected to advance the discussion about the true direction detection mechanism employed by bats. In any case, a theoretical analysis in terms of ear movement conditions would provide useful insights into why bats adopt certain ears movements and morphology.*

In addition, the direction detection mechanism for fluttering prey is important in understanding more practical situations for hunting bats. The acoustic glint generated in the CF component by a fluttering moth may have a positive influence on direction detection, but we have not yet useful investigations so far. Investigations including the acoustic glint provide more useful insights for the direction detection mechanism with spectral cues and with ILD. We will investigate the moving prey detection mechanism in our future study. Thank you for your important comments.

Comment 6:	2) Would be interesting to train the system on part of the region and test it on the rest. How animals generalize during learning is a fundamental question.

*Response:*	Thank you for this interesting comment. As you suggest, the process whereby animals acquire spatial perception is an important theme. The number of learnings for artificial intelligence seems to be much greater than that for animals. In addition, there might be no supervisor in the case of actual animal learning. Understanding the generalization capabilities of living organisms will bridge the gap to artificial intelligence. Although supervised machine learning is used as an inverse map generation tool in this study, we will investigate the ability to generalize the whole direction detection mechanism by learning a subset of directional detections in a future study.

**Comments on incorrect English notation:**

Comment 7:	There are also many places where the English should be corrected. Here are just a few examples (I could not mark them all). I strongly suggest editing the manuscript by a native English-speaking editor.
English - L72. tasks in the air,
L80 - The lateral superior olive in bats has a relatively large capacity
L103 - bat behavior always exhibits the best-benefit response.
L150 – altitude ?

*Revised point:*	Thank you for your detailed reading of our manuscript. We apologize for these errors in the English. Following your comment, we modified these sentences.

pp. 4, Lines 83–85:	*Despite the simple sensing design, i.e., only one transmitter (mouth or nose) and two receivers (left and right ears), bats accomplish precise navigation tasks such as the pursuit of prey [2, 3] and flying together with multiple conspecifics [4, 5].*

pp. 4, Lines 92–94: *The lateral superior olive in bats is larger than in other mammals [14], and acoustic localization with ILD is physically suited to less-diffractive high-frequency sound.*

pp. 5, Lines 122-124: *Although some studies measured the precise 3D pinnae motions [20], it would be difficult to understand the optimality in ears’ motion by only actual measurement behaviors.*

pp. 9, Lines 199-201: *In our model, the amplitude modulation of the echo is caused by changes in the spatial orientation of the ear.*

In addition, our revised manuscript has been completely checked by an English proof-reading service.

**Finally:**

Comment 8:	Figure legends are too short making it impossible to understand the figures.

*Revised point:*	We apologize for the unclear figure legends. We have made every effort to make the legends legible.

Comment 9:	This paper should be read and cited – fast moving bat ears create information Doppler shifts (by Mueller et al.)

*Response:*	Thank you for your recommendation. We have already cited this paper (e.g., Lines 116, 122, 563-566).

**Reviewer #2**

**Reviewer #2 Remarks:**

In this study, the authors used a theoretical approach to investigate which type of outer ear movements can facilitate sound source localization. They modeled an active listening system based on simulated pinna movements and artificial echolocation signals of a CF-bat, *Rhinolophus ferrumequinum*, and found that only specific three-axis rotational movements of the pinnae are able to facilitate accurate sound source localization in the frontal hemisphere. While the findings presented in the manuscript are generally interesting and might have the potential to advance the field of active acoustic sensing, their relevance for and applicability to natural behavior is unclear.

*Acknowledgment*:	Thank you for careful review from a neuroethological point of view. We have made every effort to revise the paper in accordance with your important comments. In particular, the Introduction and Discussion sections have been carefully revised to avoid any over-speculation regarding actual bats’ behavior. We hope you agree that our revisions are sincere.

**General Comments:**

Comment 1:	Despite the model being based on pure artificial input, the authors directly relate the model’s output to the natural behavior of bats. In my eyes, this is only valid if it can be shown that the simulated ear movements implemented in the model resemble those measured in a natural situation. What is the advantage in knowing which type of ear movement allows for maximal sound localization accuracy when it is not known if the bats are physiologically able to perform this kind of ear movement? If it is not possible for the authors to validate their findings by experimental data, I recommend to remove or at least to weaken the statements about the behavioral relevance of their findings throughout the manuscript.

*Response*:	Thank you for your important comments. As you point out, we should not attempt to identify the actual behavior employed by bats based solely on our findings, because our investigation includes several assumptions and simplifications, such as ILD detection and a simple anisotropic hearing directivity pattern. Therefore, we have completely revised the Discussion section to tone down the link between our results and bat behavior. In addition, we have added a new paragraph in the Discussion section to compare our findings with actual bat mechanisms. The revised points are described in detail in **response to** **your comments 24-33**.
While some recent studies have conducted spatial localization representation using complex ear structures based on scans of actual bats’ pinnae, we feel that it is becoming more difficult to directly understand the essential mechanisms of direction detection behind these overly complex ear structures. From the general point of view of acoustic sensing, the fundamental question is “what kind of pinnae structure and what kind of pinnae motions have a critical effect on direction detection?” To extract the essential requirements for pinnae structure and motion, we believe that it is also important to accumulate theoretical knowledge from a simplified design. In our present study, four essential motion requirements are determined in conjunction with an anisotropic hearing directivity pattern based on the ILD detection mechanism. In future work, it may be possible to reduce the motion requirements by introducing a hearing directivity pattern that is closer to that of actual bats. Moreover, simulations with a spectral cue-based direction detection mechanism have the potential to output different motion requirements. As described in the Introduction, theoretical investigations can isolate the various factors of acoustic localization and provide insights into their essential components. We hope that you agree with us about the value of theoretical investigation alongside measurements for investigating actual bats. This motivation and important findings are also described in the Discussion section (please see **response to** **your comments 24**).

Comment 2:	The authors claim that with their study, they evaluated the importance of ear movements for 3D target localization behavior in echolocating bats. It is widely accepted that bats estimate the distance of a target (i.e. target position in the depth dimension) from the temporal delay between the emitted call and the returning echo. Information on echo delay was, however, not implemented into the model in the present study, and therefore the authors’ claim is not valid. The study only allows to draw conclusions regarding target localization in 2D (azimuth/elevation).

*Revised point*:	Thank you for your comments. We apologize for the confusing expression of “3D direction detection.” As you state, depth-dimension detection with the FM component is not considered in this article. Therefore, the incorrect term “3D” has been completely removed from the revised article.
Moreover, we have clarified the description of our study in the Introduction:

pp.6, Lines 142-144: *Distance detection with the FM part is omitted from our simulations because the distance detection mechanism in bats’ echolocation is well known [7, 23].*

Comment 3:	The model generated by the authors is based on the assumption that pinna movements in bats produce a certain pattern of interaural level differences (ILDs), and that these ILDs are responsible for 3D target localization. 1) While it seems indeed to be widely assumed that pinna movements generate amplitude modulations in echoes arriving at a bat’s ears, and consequently a temporal pattern of ILDs, I am wondering whether there exists experimental evidence for this assumption. Measurements of natural ear movements in *R. ferrumequinum*, and of the effects these movements have on sounds arriving at the bat’s ear canal, have so far only shown that the ear movements produce detectable Doppler shifts, and that the time-frequency Doppler shift signatures at the bat’s ear drum encode sound source direction in an orderly fashion (Yin & Müller, 2019, Proc Natl Acad Sci U S A 116(25):12270-12274). 2) It has often been demonstrated that bats use binaural acoustic cues such as ILDs for sound localization in the horizontal plane. For sound localization in the vertical plane, however, spectral sound properties, such as notches in the echo spectrum generated by the sound filtering properties of the outer ears, are used, and to estimate target distance, bats use the time delay between emitted call and returning echo. So, what is the background for assuming that ILDs can enable target localization in elevation and in the depth dimension? Interestingly, when the ears of R. ferrumeqinum are fixed so that no ear movements could be performed by the bat, the bat is still able to localize target positions in azimuth, but not in elevation (Mogdans et al., 1988, The Journal of the Acoustical Society of America 84, 1676-1679). This indicates that ear movements in this bat may indeed be beneficial for vertical target localization. However, the underlying mechanism is unclear. It might well be possible that the bats indeed use the direction dependent Doppler shift signatures for sound localization in the vertical plane.

*Revised point*:	Thank you for your important comments. First, we apologize for using the confusing term “3D” in our manuscript. This has been completely removed. As you state, depth-dimension detection is not accomplished by the CF component. In fact, we had already described the depth-dimension detection mechanism employed by bats in the Introduction (pp. 4, Lines 106–109): “*Previous physiological and ethological studies have clarified that CF-FM bats detect the precise time interval between pulse emission and echo arrival using the FM part to measure the distance to the object accurately [7, 23].*”.

We are aware of the important article written by **Yin & Müller, 2019,** which inspired our present study. As you point out, the acoustic cues contained in the Doppler shift are important factors in investigating the direction detection mechanism. Moreover, **Yin & Müller** clearly demonstrated the direction detection performance using time–frequency Doppler shift signatures by the artificial mimicking approach.
We apologize for not introducing several important studies investigating elevation detection mechanism using key signatures other than ILD. We have added the following discussion of previous studies investigating the elevation detection mechanism based on Doppler shift signatures and hearing directivity deformation by soft ear movements:

pp. 5, Lines 112–119: *According to obstacle avoidance experiments using bats in a chamber suspended by multiple vertical and horizontal wires, the elevation angle detection performance is significantly decreased by the inhibition of both pinnae movements [27]. Elevation angle detection mechanisms have been investigated based on acoustic cues such as ILD [28, 29], the Doppler shift effect [20], and hearing directivity deformation by moving the soft material of the ear [30]. The findings indicate that the ear movements employed by bats have the function of obtaining the acoustic cues for elevation angle detection in the sound pressure domain and/or frequency domain.*

However, without measuring the auditory response of CF-FM bats while listening to echoes and moving their ears, the true mechanism of elevation angle detection cannot be judged. To date, the neural basis for the elevation angle detection mechanism of CF-FM bats has not been clearly elucidated.
Nevertheless, we apologize for not fully explaining the background behind the assumption of elevation angle detection by ILD. We have added the following text:

pp. 7, Lines 168-174: *To date, previous measurements have not demonstrated that vertical direction detection is achieved using the amplitude modulation caused by pinnae movement. However, an electrophysiological study reported that the region in space to which collicular neurons exhibit maximum sensitivity to acoustic stimuli depends on the position of the pinna and shifts predominantly in the vertical plane when the pinna is retracted manually [28]. Therefore, the simulations assumed that the ear movements cause amplitude modulation by shifting the hearing direction.*

In addition, thank you for alerting us to the important article written by **Mogdans et al., 1988**. We have cited this article in the Introduction (pp. 5, Lines 112-114). In our simulations, the azimuth angle can be detected without ear movements, as suggested in **Mogdans et al., 1988**. Based on these simulation results, we have added **Fig** **S1** to the supporting information and have added the following text to the Discussion:

pp. 25, Lines 577–582: *According to a behavioral experiment conducted on Rhinolophus ferrumeqinum, when the ears are fixed so that no movements can be performed, the bats are still able to localize the target positions in azimuth, but not in elevation [27]. Our simulations under the no-ear-movement condition confirmed similar direction detection performance (see Fig S1), suggesting that continuous ear movements contribute to the elevation angle detection.*

We agree that notches in the echo spectrum are effective cues for elevation angle detection when broadband FM pulses are adopted. In addition, it is known that FM bats that use broadband FM signals generally do not move both pinnae. In our article, we investigate CF-FM bats, which actively move both pinnae. Therefore, elevation angle detection using notches in the echo spectrum is not considered in this study.
Based on the many useful suggestions we have received from you, we intend to conduct a mathematical simulation that more accurately reflects the actual characteristics of bats. Moreover, by extending our simulation tool, we will attempt to compare the differences in ear movement requirements between Doppler-based detection and ILD-based detection.

**Abstract:**

Comment 5:	It should be clearly communicated in the abstract that in this study the effect of simulated and not natural ear movements on target localization were investigated. Furthermore, “3D” should be replaced with “2D” throughout the abstract except for the first sentence.

*Revised point*:	In accordance with your comments, the following text has been added to the abstract:

pp. 2, Lines 41–46:	*In the simulations, direction detection under rigid ear movements with interaural level differences was mathematically investigated by assuming that bats accomplish direction detection using the amplitude modulation induced by ear movements.* *In particular, the ear motion conditions required for direction detection are theoretically investigated through exhaustive simulations of the pseudo-motion of the ears, rather than simulations of the actual ear motions of bats.*

Because “2D” generally refers to the horizontal plane, we have simply removed the term “3D.”

Comment 6:	Line 33: This sentence is confusing. To detect a sound source, it is not necessary to know its distance and direction. However, this information is vital to determine the location of the sound source. I recommend to replace “detect” by “determine”.

*Revised point*:	We have modified this sentence as follows:

pp. 2, Lines 33–35:	*To determine the position of a target object, it is necessary to know the distance and direction of the target.*

Comment 7:	Line 35: The statement that ear movements enable 3D target localization is false. Ear movements are only helpful in CF bats to localize a sound source in elevation. Horizontal target localization is well possible without ear movements. Please see Mogdans et al., 1988, The Journal of the Acoustical Society of America 84, 1676-1679. Furthermore, target distance estimation is based on pulse-echo delays and should be entirely independent from ear movements.

*Revised point*:	In accordance with your comment, we have modified the text as follows:

pp. 2, Lines 35–38:	*Certain bat species that use a combined signal of long constant frequency and short frequency modulated ultrasounds synchronize their pinnae movement with pulse emission, and this behavior has been regarded as helpful for localizing the elevation angle of a reflective sound source.*

Comment 8:	Line 54: What is meant with “essential theory behind ear movements”? Do the authors refer to the mechanism or the effect of the movement?

*Revised point*:	In accordance with your comment, we have modified the text as follows:

pp. 3, Lines 61–66: *Certain bat species move both of their ears while receiving the echoes, but the control law of the effective ear movements for direction detection remains unclear. In particular, although ear movements have been investigated through practical demonstration or simulations based on actual behavioral measurements, there has been little theoretical consideration of the kinematic requirements for amplifying spatial information based on an objective viewpoint.*

Comment 9:	Line 59: The significance of pinna movements for echolocation has been revealed already some time ago, e.g. by Mogdans et al., 1988, The Journal of the Acoustical Society of America 84, 1676-1679.

*Revised point*:	In accordance with your comment, we have modified the text as follows:

pp. 3, Lines 71–73:	*This study not only theoretically supports the significance of pinnae motion in bats, but also opens up the possibility of engineering applications for active listening systems.*

Comment 10:	Line 69: There might be a better definition of ECHOLOCATION. For example, echolocation does not necessarily require ultrasound.

*Revised point*:	In accordance with your comment, we have modified the text as follows:

pp. 4, Lines 80–83:	*Echolocation describes the active acoustic sensing capability whereby the surroundings can be imaged using the echoes from pulse emissions. Bats perceive the three-dimensional (3D) environment through echolocation with high-frequency ultrasound [1].*

Comment 11:	Line 81: ILDs are indeed important for horizontal target localization in bats, but for target localization in the vertical plane, the sound filtering properties of outer ear structures are exploited (see e.g. Lawrence & Simmons, 1982, Science 218:481-483). And for target distance estimation, bats use call-echo delays. So, 3D sound localization in bats is based on a combination of ILD, echo spectrum and pulse-echo delay, and ILDs alone are only the key mechanism for horizontal sound localization.

*Revised point*:	Thank you for alerting us to this important article. We also agree that FM bats accomplish 3D localization by integrating the information of ILD, notches in the echo spectrum and a cross-correlation function.
As you mention, ILD is not the only important mechanism for 3D localization for CF-FM bats. At least, cross-correlation process is performed on FM portion to detect the depth dimension. Moreover, horizontal angle detection is accomplished by the ILD. Therefore, we have changed this sentence as follows:

pp. 4, Lines 94–95:	*Thus, the ILD encoding mechanism is regarded as one of the key properties for echo-arrival-direction detection in bats.*

Comment 12:	Line 101: What is meant by “essence of appropriate pinnae motions”?

*Revised point*:	In accordance with your comment, we have modified the text as follows:

pp. 5, Lines 120–122:	*Several previous studies have investigated the usefulness of ear motions for echo direction detection through mathematical simulations [29, 31] or practical demonstrations [20, 32], but the control law of effective ear movements remains unclear.*

Comment 13:	Line 102: While it is well possible to record precise 3D ear movements in bats with technology available today (Yin & Müller, Proc Natl Acad Sci U S A, 2019, 116(25):12270-12274), studying their effect on bat behavior is indeed less trivial. Nevertheless, recordings of natural pinna movements should at least be used to test whether the simulated ear movements used in the present study can actually be performed by the bats. What is the advantage of knowing which kind of ear movement would be ideal for target localization when the bats are not able to perform such movements due to physiological limitations? This drawback of the theoretical approach needs to be mentioned here also.

*Revised point*:	Thank you for your critical comments. As you mention, it is important to investigate the physical limitations of the pinnae muscles. In addition, it would also be helpful if we could confirm that our theoretically proposed movements are in good agreement with the actual pinnae movements of CF-FM bats. This issue will be a subject for future consideration. In accordance with your comments, we have carefully modified the Discussion to avoid any enforced estimation of the actual pinnae movements employed by bats (please see **response to** **your comments 24-33**).
As you mention, an investigation based on the precise measurements of the pinnae motions and pinnae structure is critical for understanding the direction detection mechanism of certain bat species. In particular, Yin & Müller conducted a wonderful study by measuring the *Rhinolophus ferrumequinum* and demonstrating an artificial mimicking approach.
However, to understand why bats adopt certain ear movements and clarify the essential techniques for these ear movements, theoretical analysis from a general point of view is important before determining the natural complex pinnae movements directly. In particular, our study theorized (i.e., clarified the conditions for) the control law in common with good pinnae movement patterns through exhaustive comparisons of other movement patterns.
One of our important messages is as follows:

*A more generalized expression of our results is that “in the case of a rigid ear which reproduces the simple anisotropic hearing directivity pattern, accurate direction detection is theoretically guaranteed if the ear movements satisfy the four conditions described in this paper.”(*pp. 24, Lines 552–555*)*

This is an important message from theoretical scientists to behavioral scientists. This message is more important than the five specific pinnae movements identified as good patterns. For example, our study reproduced the ear rotation by assuming *sin* and *cos* functions, but actual bats may not employ such coherent functional rotation. Our generalized message does not force such a coherent rotation. Any movement is acceptable as long as it satisfies the four conditions. Such a generalized movement suggestion is one of the advantages of our theoretical approach. We believe that such a theoretical analysis is useful for supporting a behavioral understanding because generalized theoretical requirements accept the degree of freedom necessary for ear movements.
Nevertheless, we could not compare our identified patterns with actual pinnae movements in the present study. Therefore, we have completely revised the Discussion to avoid any forcible estimation of the actual pinnae movements employed by bats (please see **response to** **your comments 24-33**).
In addition, we have added some text to the Introduction to convey our research motivation.

pp. 6, Lines 120–138:	*Several previous studies have investigated the usefulness of ear motions for echo direction detection through mathematical simulations [29, 31] or practical demonstrations [20, 32], but the control law of effective ear movements remains unclear. Although some studies measured the precise 3D pinnae motions [20], it would be difficult to understand the optimality in ears’ motion by only actual measurement behaviors. To understand the key technique for the ear movements, it is necessary to compare the direction detection performance to any other pseudo-movements of the ears.*

*In contrast, a theoretical approach allows us to evaluate various pinnae motions, including those of bats. Moreover, theoretical investigation can isolate the various factors of acoustic localization and provide insights into their essential components. For example, the unique characteristics of ear movement suited for individual acoustic cues (i.e., acoustic cues in the sound pressure domain and frequency domain) can be independently considered based on acoustic physics. Thus, theoretical analysis provides the 3D kinematic requirements of pinnae movements for accurate direction detection with and without actual bat characteristics, and generalizes this to the movement theory with suitable degrees of freedom from the general point of view of binaural listening tasks. The integration of such a pinnae control theory with physiological and behavioral findings gives an interpretation of bat behavior, and would possibly provide support for biomimetic applications.*

Comment 14:	Line 108 and following: This paragraph is misleading. Please make clear that not ear movements but only simulations of ear movements were studied.

*Revised point*:	In accordance with your comment, we have modified the text as follows:

pp. 6, Lines 139–142:	*Based on these motivations, rigid ear movements for elevation angle detection with ILD were mathematically investigated through a series of simulations. The simulations were conducted under the assumption that bats accomplish elevation angle detection from the amplitude modulation induced by ear movements in the CF part of this study.*

Comment 15:	Line 130: It is completely unclear to me where the data on amplitude modulation is coming from. Was the pattern measured with the artificial ears in the present study? Please provide information on the body of evidence showing that pinna movements in *R. ferrumequinum* generate strong amplitude modulations in the echoes impinging on the bat’s ears during echolocation. Or clarify that this is only an assumption.

*Revised point*:	Thank you for your important comments. In response to your comments, we have added the following text to clarify that we are assuming amplitude modulation.

pp. 7, Lines 168–176:	*To date, previous measurements have not demonstrated that vertical direction detection is achieved using the amplitude modulation caused by pinnae movement. However, an electrophysiological study reported that the region in space to which collicular neurons exhibit maximum sensitivity to acoustic stimuli depends on the position of the pinna and shifts predominantly in the vertical plane when the pinna is retracted manually [28]. Therefore, the simulations assumed that the ear movements cause amplitude modulation by shifting the hearing direction. Figure 1B shows a schematic diagram of the CF2 component in the echo assuming the amplitude modulation caused by pinnae motion.*

Comment 16:	Line 135: Was only the silent interval between the two echoes removed or were also the FM parts of the echoes removed? What was the reason for removing the silent interval between the echoes?

*Revised point*:	Thank you for your comments. The reasons for removing the silent interval and FM part are now described as follows:

pp. 8, Lines 180–182:	*Note that the silence between the 1st and 2nd echoes has been removed because the result is the same whether it is present or not. Thus, both signals were combined.*

pp. 7, Lines 159–160:	*Amplitude modulation calculations for the FM part were omitted so that the pure performance of direction detection could be evaluated using CF_2_.*

Comment 17:	Line 150: I am not sure if “attitude” is the correct word for spatial orientation of the ears.

*Revised point*:	In accordance with your comment, we have modified the text as follows:

pp. 9, Lines 199–201: *In our model, the amplitude modulation of the echo is caused by changes in the spatial orientation of the ear.*

Comment 18:	Line 152 and following: The directionality of hearing has been measured experimentally in R. ferrumequinum by Grinnell & Schnitzler, 1977 (J Comp Physiol A 116:63-76). Although the simulated ear directionality used in the present study roughly resembles the behaviorally measured hearing directionality, I wonder if it might be possible to implement the measured hearing directionality into the model instead of the simulated one to make the model a bit less artificial?

*Response*:	Thank you for your advice and for alerting us to this interesting article. Although we made every effort to find actual measurement data for the hearing directivity pattern of CF-FM bats, we could not find any actual data. This issue will be considered in future work.

Comment 19:	Line 162: Why 3D? The directionality of hearing is 2D, with the dimension’s azimuth and elevation.

*Revised point*:	In accordance with your comment, we removed term “3D”. Instead, we have added the text as follows:

pp. 9, Line: 212-213:	*In addition, the hearing directivity pattern forms anisotropic in the radial direction around the hearing on-axis.*

Comment 20:	Line 350: Which threshold was assumed for considering a specific detection error still to allow for accurate detection? What degree of detection error would prevent a bat from localizing a target?

*Revised point*:	Thank you for your comment. In that section, we only present an example, therefore no strict threshold is defined. Instead, for the case of a relative evaluation, we modified the text as follows:

pp. 18, Lines 415–417:	*The detection errors E[M] are 2.1° and 2.5°, indicating that relatively accurate direction detection is accomplished compared with the previous examples shown in Fig 4.*

Comment 21:	Line 392: How was “precise direction detection” defined?

Comment 22:	Line 395: What defines a “good motion pattern”?

Comment 23:	Line 401: Ah, ok. A detection error of 5° was used as threshold. This should be explained in the text and not only in the figure caption. Is there any behavioral relevance for this 5° threshold?

*Revised point*:	In accordance with your comments, we have added the following text:

pp. 20, Lines 459–463:	*If we assume that* $E\left[ M \right]<5$*° is the criterion for precise direction detection, there are 12 motion patterns (colored boxes) that achieve this level of accuracy.* *Among them, five patterns (boxes bounded by red lines) have large injection degrees, essentially indicating robust direction detection motion patterns, as discussed in the next subsection.*

**Discussion:**

Comment 24:	Discussion: Here the authors constantly make conclusions about their findings in relation to the natural behavior of bats. As long as it can’t be proven that the simulated ear movements indeed resemble ear movements performed by bats, these conclusions are not valid. I suggest that the authors in general tone down the language throughout the discussion. They may consider to drop the big statements and to focus mostly on what can indeed be concluded from their findings. The discussion is very short and could be expanded. For example, the limitations of the study should be discussed in more detail. For instance, it could be mentioned that an actual bat ear is a flexible structure not a rigid plane as simulated in the model. During natural movements, the ear is likely not only rotated but also deformed and bended in itself, which might have a strong additional effect on the hearing directionality.

*Revised point*:	Thank you for your important comments. We apologize for forcibly estimating the actual pinnae movements based on our simulation results despite not confirming the actual behavior of bats. We have improved and extended the Discussion in accordance with your useful advice. In particular, we have added a new paragraph to describe the limitations of our research at this time:

pp. 24, Lines 556–572: *In our simulations, a simple anisotropic directivity pattern was assumed for hearing. However, actual measurements of bats have shown that the hearing directivity pattern is more complex in periphery regions [17, 18, 30, 34]. Another measurement study showed that the hearing directivity pattern of bats is deformed by the bending of the soft material of the ears [30]. Such a complex hearing directivity pattern has not been investigated in the present study. There is the possibility to deregulate the ear motion conditions by implementing more precise hearing directivity pattern in bats. If so, this would provide a better understanding of the directivity formation effect in bats’ pinnae. From another perspective, it has been suggested that the ear movements produce detectable Doppler shifts, with the time-frequency Doppler shift signatures able to encode the sound source direction in an orderly fashion [20]. If we clarify the necessary conditions of ear movements for direction detection with Doppler shift signatures using a similar analysis framework, we could compare the differences in motion conditions between ILD-based and Doppler shift-based direction detection. Such a comparison could be expected to advance the discussion about the true direction detection mechanism employed by bats. In any case, a theoretical analysis in terms of ear movement conditions would provide useful insights into why bats adopt certain ears movements and morphology.*

In addition, the first paragraph of the Discussion has been revised to avoid the forcible estimation of the actual pinnae movements. In particular, our simulation assumptions and theoretical findings are clearly expressed as follows:

pp. 24, Lines 548–555:	*In this study, rigid ear movements for direction detection with ILD were mathematically investigated by assuming the amplitude modulation induced by ear movements. Our theoretical investigation suggests that only certain ear motions consisting of three-axis rotations accomplished direction detection accurately (Figs 8 and 10) and robustly (Fig 9). A more generalized expression of our results is that “in the case of a rigid ear which reproduces the simple anisotropic hearing directivity pattern, accurate direction detection is theoretically guaranteed if the ear movements satisfy the four conditions described in this paper.”*

Moreover, in accordance with **your comments 25 and 30**, incorrect expressions have been removed or changed. Please see **response to your comments 25 and 30**.

Comment 25:	Line 483: This sentence includes several statements about bat behavior for which no evidence exists. 1) Why do the authors assume that the ear movements are performed by bats intentionally? It might as well be possible that the ear movements are physiologically coupled to the motor act of call production. 2) When was shown that ear movements are used by bats for target localization in 3D? To my knowledge, it has been clearly demonstrated by previous studies that ear movements in CF-FM bats are essential for target localization in elevation but not for target localization in azimuth or in the depth dimension. 3) Why do the authors assume that ear movements are energetically demanding?

*Revised point*:	Thank you for your important comments. We apologize for this incorrect and arbitrary sentence. We have removed the following text.

*“In the real world, bats intentionally employ rapid ear motions for 3D localization, despite the high energy costs, suggesting that they provide significant benefits in the process of echolocation.”*

Comment 26:	Line 488: simulated motion patterns.

*Revised point*:	In accordance with your comment, we have modified the text as follows:

pp. 24, Lines 574–576:	*In contrast, our study has considered the theoretical basis for these ear motions by evaluating exhaustive simulated motion patterns.*

Comment 27:	Line 491: 2D instead of 3D.

*Revised point*:	In accordance with your comment, we have modified the text as follows:

pp. 25, Lines 582–584:	*The results of general case analyses (Fig 10) show that three-axes rotations are necessary for azimuth and elevation direction detection (i.e., those not including the pairing name 0).*

Comment 28:	Line 502: What message do the authors want to convey with this sentence?

*Revised point*:	In accordance with your comment, we have modified the text as follows:

pp. 26, Lines 597–598:	*Such a theoretical consideration is expected to provide the useful control design of the artificial ears’ motion for active listening systems in the future.*

Comment 29:	Line 505: In total 216 different simulated ear movements were tested. So, it should read “five out of 216 motion patterns”.

*Response*:	In the total movement pattern simulations without antiphase motion in pitch angle, fourteen out of 216 motion patterns produced accurate and robust direction detection. Therefore, “five out of 216 motion patterns” is incorrect.

Comment 30:	Line 506: No! This does not suggest that the bats select one of these five motion patterns. First, it needs to be proven that the 3D ear motion pattern of bats indeed resembles one of the five simulated patterns. And even if this would be the case, this does not show that the bats select this type of motion. They could for example due to physiological limitations only be able to perform this certain pattern of ear movement.

*Revised point*:	Thank you for your comment. As you state, it is too aggressive to estimate the actual pinnae movements based on our simulation results without confirming the actual behavior of bats. Therefore, we have changed this sentence as follows:

pp. 26, Lines 606–608:	*This pattern may be easier to implement in artificial active listening systems using ILD codes than the other four patterns.*

Comment 31:	Line 508: Which surgical plane? Do the authors mean the midsagittal plane?

*Revised point*:	Yes, we apologize for this error. We have modified the text as follows:

pp. 26, Lines 601–602:	*It is plausible to assume that the motions of the left and right ears are mirror-symmetric with respect to the midsagittal plane.*

Comment 32:	Line 513: If some support for this hypothesis could be presented, the paper would be much stronger. When watching the slow-motion video clip of Hipposideros ear movements provided with the paper by Yin & Müller (http://movie-usa.glencoesoftware.com/video/10.1073/pnas.1901120116/video-1), it seems as the ear movements consist only of a combination of yaw-axis and pitch-axis but not of roll-axis rotations.

*Revised point*:	Thank you for your kind advice. We have viewed this movie repeatedly. Our five ear movements included the temporal fixed pattern of roll axis rotation. However, we currently have no quantitative means of comparing our findings with this video clip. Therefore, this text has been completely modified as described in our response to **comment 30.** In our simulation results, two-axis rotation patterns are included. Therefore, the following sentence has been added to the Discussion.

pp. 25, Lines 584–587:	*However, all three-axes do not necessarily need to rotate continuously in time, so two-axes temporal rotation patterns were also included in the movement patterns that accomplish accurate direction detection (i.e., cases including the pairing name* $\bar{\boldsymbol{CONST}}$*).*

Comment 33:	Line 525: Well, a movement consisting of rotations along three axes, could in my eyes be considered complicated.

*Revised point*:	Thank you for your comment. Our proposed index (degree of injection) is simply a generalized mathematical evaluation function. Therefore, it is possible to evaluate every ear motion, regardless of whether three-axis rotation is used or not. In this sentence, we mention the usefulness of our original index in evaluating the localization performance. Therefore, three-axis rotation is not essentially related to this message.

**Figures:**

Comment 34:	Figure 1: It should be mentioned in the figure caption that panel B already shows the output of the model, and which exact movement produced this specific pattern of amplitude modulation and resulting ILD pattern. Why is the modulation pattern for the forward movement different from the backward movement? Shouldn’t both patterns be congruent for each ear? When the right ear is moved forward, it is at a certain point in time aligned with the beam axis of the echo, which yields maximum amplitude. Given that the position of the bat and the target doesn’t change between the forward and the backward movement, the signal envelope should show a convex pattern also for the backward movement.

*Revised point*:	We apologize for inserting the incorrect time amplitude signals in this rough sketch. We have modified the figures to show the actual simulated signals. In addition, the ear movement condition and the angle at which the reflective object is located have been added to the caption as follows:

pp. 8, Lines 185–192:	*Fig 1. Pulse emission and reflective echo patterns.
(A) Typical time-frequency structure of the echolocation pulse emitted by Rhinolophus ferrumequinum nippon. (B) Schematic diagram of amplitude modulation of CF_2_ component in the echo caused by pinnae motions. In this example, the left and right ear movements were assumed to exhibit anti-phase motions in pitch angle over* $\pm$*15°. The hearing axis in the right ear rotates down-forward and then up-backward, while that in the left ear rotates inversely. The amplitude-modulated echo was simulated by assuming the object has an elevation angle of 30°.*

Comment 35:	Figure 2: In panel B, the red dot with the word microphone next to it is confusing. Does the red dot represent the target in relation to the microphones? The authors may consider to choose a different color for the representation of the microphones and the target. In the figure caption it should be mentioned that the red dots indicate the position of the microphones.

*Revised point*:	Thank you for your comment. The color of the target object in panel A has been changed to green. In addition, the caption has been modified as follows:

pp. 9, Lines 218–224:	*Fig 2. Schematic diagram of model setup.
(A) Direction of the target expressed by azimuth angle θ and elevation angle φ. (B) Positions of the two directional ears with spacing d. Each red dot indicates the position of an omnidirectional microphone. Each ear consists of four omni-directional microphones, where* $\delta_{y}$ *and* $\delta_{z}$ *are the horizontal and vertical spacings of each microphone. (C) Hearing directivity pattern of the ear. (D) Three axes (roll, pitch, yaw) fixed to the directional ear and corresponding orthonormal basis* ${[\boldsymbol{n}}_{roll},\boldsymbol{n}_{pitch}, \boldsymbol{n}_{yaw}]$*.*

Comment 36:	Figure 4: Labeling of panels (A1 - A3, B1 – B3) is missing.

Figure 5: Labeling of panels (A1 – A3, B1 – B3) is missing.

*Revised point*:	We apologize for this incorrect labeling. We have corrected the labeling in Figs 4 and 5.

Comment 38:	Figure 9B: x-axis label = time [sec], y-axis label = ILD [dB SPL]

*Response*:	Thank you for your comment. However, the interaural level difference is not the same as the absolute sound pressure level. Therefore, the y-axis label has not been changed.
