# Supplementary material for: Theoretical investigation of active listening behavior based on the echolocation of CF-FM bats
Source: PLoS Comput Biol. 2022 Oct 7;18(10):e1009784. doi: 10.1371/journal.pcbi.1009784 (PMC9581360; doi:10.1371/journal.pcbi.1009784)
Supplement: Attachment — Submitted filename: response_letter_2nd_rev.docx [file pcbi.1009784.s007.docx]

*PLOS Computational Biology*

August 8, 2022

Dear Editor,

Re: Resubmission of manuscript reference No. PCOMPBIOL-D-21-02303

Please find attached a revised version of our manuscript “Theoretical investigation of active listening behavior based on the echolocation of CF-FM bats,” which we would like to submit for consideration for publication as an Original Article in *PLOS Computational Biology.*

The comments from the reviewers were highly insightful and enabled us to greatly improve the quality of our manuscript. The following pages present our point-by-point responses to each of the comments of the reviewers.

We hope that the revisions in the manuscript and our accompanying responses will be sufficient to make our manuscript suitable for publication in *PLOS Computational Biology.*

We look forward to hearing from you at your earliest convenience.

Yours sincerely,

Yasufumi Yamada (Corresponding author)

Program of Mathematical and Life Sciences, Hiroshima University.

C-623, Department of Sciences,1-3-2 Kagamiyama, Higashi-Hiroshima, Japan

E-mail: yasufumi.yamada1@gmail.com

**Editor Remarks:**

The reviewers are agreed that the paper is of interest, and appropriate for PLOS Computational Biology, but have substantial required changes. I therefore agree with their recommendation to request Major Revisions. Please pay attention to the reviewers' feedback. This includes fixing the data availability issue.

*Acknowledgment*:	We would like to thank the editor and reviewers for taking the time to examine our manuscript. We have made a significant effort to revise the manuscript according to the various comments made by the reviewer2. We hope that our revised paper is suitable for publication in your esteemed journal. Our responses to the individual comments of the reviewer 2 can be found below.

**Reviewer #2**

**Reviewer #2 Remarks:**

I thank the authors for the detailed replies to the reviewers’ comments. The revised manuscript is of significantly increased quality. However, some issues still remain. 1) Some parts of the manuscript are still hard to understand due to the incorrect usage of the English language. I highly recommend to take advantage of a professional language editing service.

*Acknowledgment*:	Thank you for your careful and generous review of our manuscript. We have acquired significant behavioral knowledge and identified many important issues to consider in further investigations. We have made every effort to revise the paper in accordance with your important comments. In particular, we carefully revised the English and have employed an English proofreading service. Moreover, the discussion sections have been carefully revised following your advice. We hope that our revisions meet with your approval.

**General comments:**

Comment 1:	2) I feel that the replies to the reviewers’ comments, which the authors have provided in the response letter, are often much more detailed (and helpful) than the changes that have been made to the manuscript itself. Many of the explanations in the response letter should also be included in the manuscript. For example, both reviewers raised questions, which the authors, as they state in the response letter, want to tackle with future experiments. This should be included in the manuscript, for example within a paragraph “Future Directions” at the end of the discussion.

*Response:*	Thank you for careful reading of our response letter. We apologize for not fully including our responses in the main manuscript. Based on the previous response letter, the discussion section has been greatly enriched. We hope our additional discussion meets with your approval.
In accordance with your comment, we have added some sentences on future work at the end of the discussion section:

pp.28, Lines 648-669:	*There are still many things to consider about the direction detection mechanism employed by bats. For example, the direction detection mechanism for fluttering prey is important in understanding more practical situations for hunting bats. The acoustic glint generated in the CF component by a fluttering moth [38] may have a positive influence on direction detection, but we have not yet performed any useful investigations to clarify this. Investigations including the acoustic glint would provide more useful insights into the direction detection mechanism with spectral cues and with ILD. In another perspective for future work, bat species who use only broadband FM signals accomplish direction detection without fast ear movements. For these echolocating bats, the spectral filtering function due to the interference of the echoes on the pinnae and tragus surfaces are regarded as the most important feature because ultrasound of various wavelengths provides rich information of every spectral strength [30, 39]. However, we have not yet investigated the direction detection mechanism based on spectral cues.
We must also consider the difference between animal intelligence and artificial intelligence. The process whereby animals acquire spatial perception is an important theme. The number of trainings for acquiring the spatial perception on artificial intelligence seems to be much greater than that for animal intelligence. In addition, it is more difficult to identify a supervisor in the case of actual animal learning. Understanding the generalization capabilities of living organisms will bridge the gap to artificial intelligence. Although supervised machine learning is used as an inverse map generation tool in this study, we will investigate the ability to generalize the whole direction detection mechanism by learning a subset of directional detections in a future study.*

*Response:*	In addition, the following discussion has been added to aid in understanding the significance of this study.

pp.24, Lines 557-581:	*An investigation based on precise measurements of the pinnae motions and pinnae structure is critical for understanding the direction detection mechanism of certain bat species. In particular, Yin & Müller measured the precise pinnae motions of Rhinolophus ferrumequinum [20] and demonstrated the spatial perception system by an artificial mimicking approach [36]. From the general perspective of acoustic sensing, the fundamental question is “what kind of pinnae structure and what kind of pinnae motions have a critical effect on direction detection?” To extract the essential requirements for the pinnae structure and motion, we believe that it is also important to accumulate theoretical knowledge from simplified design simulations. In particular, by assuming that ear movements create useful amplitude modulations in the echoes, our study has clarified the conditions for the control law in common with better movement patterns for direction detection through comparisons of various simulated ear movements.
One of our important messages is that, in the case of a rigid ear that reproduces the simple anisotropic hearing directivity pattern, accurate direction detection is theoretically guaranteed if the ear movements satisfy the four conditions described in this paper. This is an important message from theoretical scientists to behavioral scientists, and is more important than the five specific simulated ear movements identified as less detection error movement patterns. For example, our study reproduced ear rotation by assuming sine and cosine functions, but actual bats are unlikely to employ such coherent functional rotation. Our generalized message does not force such a coherent rotation. Any movement is acceptable, as long as it satisfies the four conditions. The suggestion of such a generalized movement is one of the advantages of our theoretical approach. We believe that such a theoretical analysis is useful for supporting a behavioral understanding because generalized theoretical requirements accept the degree of freedom necessary for ear movements.*

Comment 2:	3) It is still not clear to me what the assumption that ear movements produce amplitude modulations in the echoes impinging on the bats ears is based on. The authors cite an electrophysiological study demonstrating that spatial sensitivity of midbrain auditory neurons shifts in the vertical plane when the orientation of the pinna is changed. However, this study does not show that amplitude modulations are responsible for the shifts in spatial tuning of the neurons, and also does not prove that the pinna movements produce amplitude modulations in the echoes. The authors should explain in detail why they assume that for example the ear movement shown in Fig. 1B produces a maximum modulation depth of 50% of the echo amplitude. Is the maximum value of modulation depth equal for all different simulated ear movements? What is this maximum value based on? Was it measured or just assumed? Please clarify this in the manuscript.

*Response:*	Thank you for important comments.
As you have stated, the physiological encoding mechanism from amplitude modulation to spatial information has not yet been clarified. This fact is clearly indicated in the paper as follows:

pp.7, Lines 165-166:	*To date, previous measurements have not demonstrated that vertical direction detection is achieved using the amplitude modulation caused by pinnae movement.*

To address your comment asking “why do you assume the pinna movements produce amplitude modulations in the echoes,” we have added the following sentences:

pp.7, Lines 170-175:	*According to recent research on acoustic simulation considering the detailed pinnae shape and movements of Rhinolophus ferrumequinum Nippon, it was suggested that the pinna movements change the hearing directivity [34]. From the perspective of acoustic physics, it is natural that the amplitude of the echoes is modulated if the hearing directivity changes while receiving the echoes. Therefore, the simulations assumed that the ear movements cause amplitude modulation by shifting the hearing direction.*

*Response:*	We believe this assumption is logically correct based on acoustic physics. How do bats receive constant amplitude echoes when the hearing directivity changes?

We have clarified that Fig 1B is based on the simulations as follows:

pp.8, Lines 176-180:	*Fig 1B shows a schematic diagram of the CF2 component in the simulated echo assuming that amplitude modulation is caused by the virtual pinnae motion. The amplitude modulation depth and modulation pattern changes in response to the virtual pinnae motion. The detailed setup for determining the hearing directivity characteristics of the virtual pinnae is described in the next section.*

Comment 3:	4) Throughout the manuscript and supplementary material there are still instances where the word “attitude” is used to describe the spatial orientation of an ear. This should be rewritten.

*Response:*	Thank you for your advice. We have carefully checked and changed the word from “attitude” to “orientation.” (Lines, 204, 234, 235, 799).

**Line-specific comments:**

Comment 1:	Line 32: The term “three-dimensional” is only used once within the abstract. Therefore, the abbreviation “(3D)” does not need to be introduced in line 32.

*Response*:	We apologize for this unnecessary abbreviation and have removed it from the revised paper (page 2, line 32).

Comment 2:	Line 44: … amplitude modulation in the echoes caused by ear movements.

*Revised point*:	We have modified this sentence as follows:

pp.2, Lines 41-44:	*In the simulations, direction detection under rigid ear movements with interaural level differences was mathematically investigated by assuming that bats accomplish direction detection using the amplitude modulation in the echoes caused by ear movements.*

Comment 3:	Line 47: “allow for” instead of “accomplish”.

*Revised point*:	We have modified that according to your comment. (pp.2, Line 48)

Comment 4:	Line 50: “provide the conditions for ear motions to ensure accurate and robust direction detection,” can be removed entirely, since this part of the sentence is repeated in the following part of the sentence. It is sufficient to write: “In addition, we suggest that simple shaped hearing directionality and well-selected uncomplicated ear motions …”

*Revised point*:	We have completely removed that sentence according to your comment. (pp.2, Lines 50)

Comment 5:	Line 62: “control law” sounds a bit awkward. Why not “… but the precise type of ear movements that facilitate direction detection…”

*Revised point*:	We have modified this sentence as follows:

pp. 3, Lines 60–62:	*Certain bat species move both of their ears while receiving the echoes, but the precise type of ear movements that facilitate direction detection remains unclear.*

Comment 6:	Line 68-69: “The theory suggests that certain ear motions might enable highly accurate direction detection that is robust to observation errors.”

*Revised point*:	We have modified that according to your comment. (pp.3, Lines 67-68)

Comment 7:	Line 81: Not all echolocators use pulses as sonar signals. Maybe “sound emissions” is a better term.

*Revised point*:	We have modified that according to your comment. (pp.4, Line 80)

Comment 8:	Line 95 and throughout the ms: “echo-arrival direction detection” sounds strange. Maybe use “echo source direction detection” instead?

*Revised point*:	We have modified that according to your comment. (pp.4, Line 94,97 and pp.5, Line 101)

Comment 9:	Line 112-114: This sentence is hard to understand. Maybe rewrite to: “Obstacle avoidance experiments using bats flying in a chamber that contained multiple vertically and horizontally suspended wires showed that elevation angle detection performance is significantly decreased when bats are prevented from moving their pinnae [27].”

*Revised point*:	Thank you for suggesting this improved wording. We have modified this sentence as follows:

pp. 5, Lines 111–113:	*Chamber experiments investigating the obstacle avoidance flight of bats have shown that elevation angle detection performance is significantly decreased when bats are prevented from moving their pinnae [27].*

Comment 10:	Line 122: Please replace “control law” by e.g. “precise nature”.

*Revised point*:	We have modified that according to your comment. (pp.5, Lines 119-121)

Comment 11:	Line 124: “… the optimality in ears’ motion by physiological measurements only.”

*Revised point*:	We have modified the sentence as follows:

pp.5, Lines 121-123	*Although some studies have measured the precise 3D pinnae motions [20], it would be difficult to understand the optimality of the ear motions by physiological measurements alone.*

Comment 12:	Line 132-135: This is a very complicated sentence and hard to understand. Maybe the authors might consider to split the sentence into two shorter sentences.

*Revised point*:	Thank you for your comments. We have shortened this sentence as follows:

pp. 6, Lines 131–132:	*Thus, theoretical analysis provides the 3D kinematic requirements of pinnae movements for accurate direction detection through binaural listening tasks.*

Comment 13:	Line 136-137: “The integration of such a pinnae control theory with physiological and behavioral findings might give an interpretation of bat behavior, …”

*Revised point*:	We have modified that according to your comment. (pp.6, Lines 132-134)

Comment 14:	Line 139-142: “Based on these motivations, rigid ear movements for echo source detection based on ILD were mathematically investigated through a series of simulations. The simulations were conducted under the assumption that bats accomplish echo source detection by using the amplitude modulation induced by ear movements in the CF part of the echoes.”

Comment 15:	Line 143: “Distance detection of echo sources with the FM part of the echoes is omitted from …”

Comment 16:	Line 144-146: “In particular, simulations of different ear motions were analyzed to identify the nature of ear motions suited best for sound source detection.”

*Revised point*:	According to your comments, we have modified those sentences as follows:

pp. 6, Lines 136–143:	*Based on these motivations, rigid ear movements for echo source detection based on ILD were mathematically investigated through a series of simulations. The simulations were conducted under the assumption that bats accomplish echo source detection by using the amplitude modulation induced by ear movements in the CF part of the echoes. The distance detection of echo sources with the FM part of the echoes was omitted from our simulations because the distance detection mechanism in bats’ echolocation is well known [7, 23]. In particular, simulations of different ear motions were analyzed to identify the nature of ear motions that are suited to sound source detection.*

Comment 17:	Line 180-182: This is not a very convincing argument for removing the gap between the two successive echoes. If the results are the same for simulations including the gap and simulations excluding the gap, I would prefer seeing the results with the gap present over seeing results of simulations without a gap between the two echoes. Removing the silent interval between echoes generates a very unnatural acoustic stimulus, which will never be available to a real bat. By doubling the duration of the CF part of an echo, a bat would be provided with an unnaturally long time to extract spatial information from the echo, which in theory could amplify sound source localization. To make the results more comparable to a natural situation, the input to the model should be kept as naturalistic as possible.

*Response*:	Thank you for your important comments. As you have stated, in the case of actual echolocation, there is a silent interval between the 1^st^ and 2^nd^ echoes. However, this silent interval does not contribute to the direction detection from a computational perspective. Therefore, we remove the silent intervals in our model. Thus, we have modified the text as follows:

pp. 8, Lines 184–186:	*Note that the silent interval does not contribute to the direction detection from a computational perspective. Therefore, we combined the 1st and 2nd echoes in our model, as indicated in Fig 1B.*

Comment 18:	Line 202-207: Based on this description, I am still confused whether this was a real setup or a simulation. The abstract reads as if physical microphones have been used to measure echoes and their output has been used as input for the model. However, I assume this was not the case. Maybe this is clear to computational scientists, but for readers not so familiar with mathematical models, this paragraph should be somehow rewritten in a way that makes clear that there was no physical microphone or any sound recordings were involved. Maybe it will be helpful to write “simulated ears” instead of “directional ears”?

*Revised point*:	Thank you for your kind advice. To clarify that these simulations were conducted without practical measurements, the important terms “virtual” and “simulated” have been added in this paragraph (lines 176–179, 192, 200, 205, 210, 227).
In this study, the traditional engineering method of beamforming was applied to our virtual ears. Therefore, we believe that “directional ear” is a more appropriate term than “simulated ear.” Thus, to clarify that this is a simulation study, we have modified the corresponding sentences as follows:

pp. 9, Line: 212-213:	*In our model, the amplitude modulation of the echo is caused by changes in the spatial orientation of the virtual ear. Fig 2B shows a schematic diagram of the left and right ears and a speaker when all materials are directed in front of the bat (positive direction of x-axis). In the engineering field, beamforming is a popular method of synthesizing the signals from multiple microphones, as seen in phased array systems [35]. Following this method, four omni-directional microphones were placed at the vertices of a rectangle to construct a virtual ear.*

Comment 19:	Line 212-213: I don’t understand this sentence.

*Revised point*:	Thank you for pointing out. Since this sentence is not very important in this article, we removed following terms,

pp. 9, Line: 211:	*In addition, the hearing directivity pattern forms anisotropic in the radial direction around the hearing on-axis.*

Comment 20:	Line 256-257: To simplify this sentence, the part after the comma can be removed: “In this section, we prepare a general mathematical framework for describing and evaluating the active sensing process.”.

*Revised point*:	We have modified that according to your comment. (pp.11, Lines 260-261)

Comment 21:	Line 381-382: In my opinion, it is not correct to state that bats do not perform complex ear movements. I as a human being who is not able to move its ears at all, consider the ear movements performed by bats as highly complex.

*Revised point*:	We apologize for this misleading expression. We actually meant that higher-order Fourier modes are not included in each roll, pitch, and yaw component. By combining the first-order Fourier modes in the roll, pitch, and yaw components, complex ear movements can be represented. In accordance with your comment, we have changed the wording as follows:

pp. 16, Line: 383-386:	*In our model, the periodic motion in each roll, pitch, and yaw component is assumed to be the 0th and 1st Fourier modes. Note that by combining each roll, pitch, and yaw movement, the various ear movements can be represented.*

**Discussion:**

Comment 22:	Line 549-551: “In this study, simulations of rigid ear movements for direction detection with ILD were mathematically investigated by assuming that ear movements create useful amplitude modulations on the echoes perceived by bats.”

*Revised point*:	According to your comment, we have modified that sentence as follows:

pp.24, Lines 552-554	*In this study, mathematical models of rigid ear movements for direction detection with ILD were investigated by assuming that ear movements create useful amplitude modulations in the echoes.*

Comment 23:	Line 552: “… three-axis rotations can be useful for accurate echo direction detection.”

*Revised point*:	Thank you for this comment. This important message is derived from a completely theoretical analysis based on the evidences of Figs 8–10. We have clarified this message by using the phrase “simulated ear” as follows:

pp. 24, Line: 554-556:	*Our theoretical investigation clarified that only certain simulated ear motions can achieve echo source direction detection accurately (Figs 8 and 10) and robustly (Fig 9).*

Comment 24:	Line 559: remove the word “measurement”

We have modified that according to your comment. (pp.26, Lines 584-586)

Comment 25:	Figure 2A: What is indicated by the two grey dots located at the circle in the horizontal plane

*Revised point*:	Thank you for your comment. The gray dots indicate the histories of azimuthal and elevation rotations of the target object (green ball). In accordance with your comment, we have added historical arrows (shown in orange) for these two rotations and changed the gray dot sizes to be same as the green dot.
